# The 2018 Censo Nacional de Población y Vivienda: the Good, the Bad and the Ugly

**DOI:** 10.25100/cm.v50i1.4335

**Published:** 2019-03-30

**Authors:** Julian Santaella-Tenorio

**Affiliations:** 1 Adjunct Professor. Escuela de Salud Pública . Universidad del Valle. Cali, Colombia; 2 Post-doctoral fellow. Department of Population Health, School of Medicine, New York University, NYU, New York, USA

Preliminary results from the 2018 Census by the National Administrative Department of Statistics (DANE) have shown interesting news for Colombia, the major one probably being that instead of the projected 50 millions of habitants expected in 2018 in the Census 2005 projections, Colombia’s population in 2018 was 45.5 million (DANE 2018). Although, the differences in population projections vs. censuses totals have been described across countries in Latin America ^1^, Colombia seems to be a country with a major gap. Knowing how many people live in the country is important for governmental planning, public spending, and to set up goals according to public needs. Therefore, these news from the 2018 Census were received with caution among different sectors.

This is not the first time something like this happens in the country as projections from the 1993 Census also did not match the population estimates from the 2005 Census, with the projection being short by 3,150,552 individuals. Back then, the difference between the 1993 projections and the 2005 Census results was attributable to differences in collection methods used in 2005 and an initial overestimation of the 1993 census population (1,051,965 individuals), an overestimation of natality rates, and also an underestimation of emigration rates (mainly for women of reproductive age) ^2^. Similarly, it possible that the current differences between 2005 projections and the 2018 Census results were caused by methodological issues, but also social changes, such as the reduction in natality rates linked to economy improvements and a growing middle-class.

An important problem with differences in projected vs. recent Census data is that trajectories of economic or health indicators used to describe progress or regressions in these sectors become challenged by them. An issue is that there is a jump in the trajectory of disease rates when using the 2018 population estimate instead of the 2005 projected for 2018. This jump is explained by the change in the denominator used to calculate the rate, not by an actual increase in the number of cases. A good example is the change in the homicide rate in Cali, where the 2005 Census projections differed from the 2018 census estimates by 28.7% (for Colombia, projections were over the 2018 estimate by 9.5%) ^3^. The population for Cali in 2018 with the 2018 Census was estimated to be 1.9 million, which is even lower than the population estimated for 2005 with the 2005 Census (2.1 million) ^3^.

**Figure 1:**
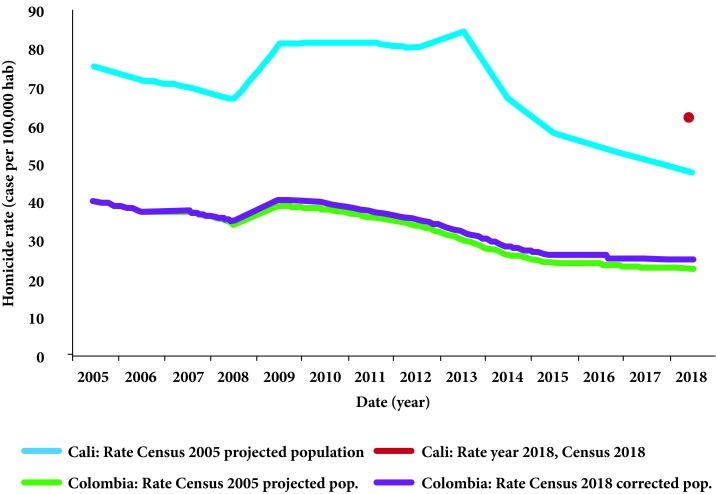
Homicide rate in Cali and Colombia using the 2005 Census population projections and the corrected population estimates based on 2018 differences.*The correction of population estimates for the Country was based on an assumed yearly linear increase in the differences between the 2005 projections and the 2018 Census population estimates for 2018. The figure shows no major changes in the trend for the homicide rate in Colombia when using the projected vs. the new calculated yearly populations.

When the population from the 2018 Census is used, the homicide rate in Cali in 2018 changes from 47.68 per 100,000 (using 2005 projections) to 61.37 per 100,000 (Figure 1). Despite the continuous decrease in the homicide rate from 2013 to 2018, this would mean that Cali would now rank at the top of the list of cities with highest homicide rates in the world ^4^ ^,^ ^5^, passing from the 26^th^ to 28^th^ places in the list to be ranked between the 10^th^ to 15^th^ places. 

But, were the 2005 population projections for Cali overinflated or was the population severely underestimated in the Census 2018? The 2018 Census results suggest that there was a reduction in Cali’s total population in the last 13 years. However, it is not likely that the population in Cali shrank overtime. Data from DANE on the annual number of live births and the number of fatalities from 2005 to 2018 indicate that the population in 2018 should have been around 2.3 million ^6^. Data on affiliations to the to health system also suggest that the population in Cali continued increasing since 2005 ^7^. Given the expected population growth in the last decade in Cali and the evidence from other data sources, it is possible that issues in the implementation of the 2018 Census in Cali resulted in biased estimates of the population. 

As the DANE analyses the reasons behind the lower population estimate in Cali and other municipalities in Valle del Cauca as well as in other departments and tries to resolve the inconsistency, researchers and policy makers are eager to know which denominators to use in their analyses. Knowing these is key to a better understanding of disease trends and the impact of policies aiming at reducing population risks.
