# Supplementary material for: The 2018 Censo Nacional de Población y Vivienda: the Good, the Bad and the Ugly
Source: Colomb Med (Cali). 2019 Mar 30;50(1):11–2. doi: 10.25100/cm.v50i1.4335 (PMC6536045; doi:10.25100/cm.v50i1.4335)
Supplement: Related texts

# Editorial: Censo Nacional de Población y Vivienda 2018: lo Bueno, lo Malo y lo Feo

Los resultados preliminares del Censo 2018 por el Departamento Administrativo Nacional de Estadística (DANE) han mostrado interesantes noticias para Colombia, la mayor de todas ellas, tal vez, que la población colombiana en 2018 no llegaba a los 50 millones proyectados en el Censo de 2005, sino solo a 45.5 millones (DANE 2018). Aunque es común que en otros países de Latinoamérica las proyecciones de censos pasados no concuerden con las nuevas estimaciones censales ^1^, Colombia al parecer tiene una de las mayores diferencias entre estimaciones en la región. Saber cuántas personas habitan en el país es importante para la planeación gubernamental, la planeación del gasto público, y la identificación de metas acorde a las necesidades de la población. Por esta razón, los resultados del Censo 2018 han sido tomados con preocupación por diferentes sectores. 

Esta no es la primera vez que algo así pasa en el país, ya que la proyección del Censo de 1993 tampoco estuvo cerca de la estimación poblacional del Censo 2005, siendo menor en 3,150,552 habitantes. La diferencia entre la proyección del Censo de 1993 con la estimación del 2005 al parecer fue debida a diferencias en los métodos de recolección de la información usados en 2005, una sobreestimación inicial de la población en 1993 (1,051,965 habitantes), una sobreestimación de las tasas de natalidad, y también una subestimación en las tasas de emigración (principalmente para mujeres en edad reproductiva) ^2^. Similarmente, es posible que la diferencia actual entre la proyección del 2005 y la estimación del Censo de 2018 se haya generado por diferencias metodológicas, así como por cambios sociales, tales como la reducción de las tasas de natalidad ligadas a cambios económicos y el crecimiento de la clase media.

Un problema importante de estas diferencias entre proyecciones y nuevas estimaciones es el desafío que generado para comprender las trayectorias económicas o de indicadores de salud usados para describir el progreso o retrocesos en estos sectores. Al usar una nueva población (ej., del Censo 2018) para calcular la tasa de enfermedad se genera un salto en la trayectoria para el año 2018, que es explicado por el cambio del denominador y no por un cambio en el número de casos. Un buen ejemplo de esto es la tasa de homicidio en Cali, donde la proyección del Censo 2005 sobreestimó en un 28.7% la población en el Censo de 2018 (en Colombia la sobreestimación fue del 9.5%) ^3^. El estimado de la población de Cali basado en el Censo de 2018 fue de 1.9 millones, valor que incluso es menor a la población estimada en el 2005 por el Censo de 2005 ^3^. 

Cuando la población del Censo de 2018 es usada, la tasa de homicidio en Cali en el 2018 cambia de 47.68 por 100,000 (al usar la proyección de 2005) a 61.37 por 100,000 (Fig. 1). A pesar de la continua disminución de la tasa de homicidio desde 2013, este cambio en 2018 haría que Cali pasara a ser una de las ciudades con mayores tasas de homicidio en el mundo ^4^^,^^5^, pasando de los puestos 26 a 28 a estar entre los 10 a 15 de la lista.

**Figura 1:** Tasa de homicidios en Cali y Colombia al usar las proyecciones del Censo 2005 y la población corregida basada en la diferencia entre la población en 2018 proyectada con el Censo 2005 y la estimación del Censo de 2018. La corrección de los estimados de población para el país se basa en un crecimiento lineal de la diferencia la población en 2018 entre las proyecciones de 2005 y la estimada con el Censo de 2018. La figura muestra que no han cambios importantes en las trayectorias de la tasa de homicidios en Colombia cuando se usa la tasa basada en la proyección y la calculada con base en las diferencias de población en 2018.

¿Sin embargo, estaba inflada la proyección de la población de Cali basada en el Censo de 2005? ¿O subestimada la población en el Censo de 2018? Aunque los resultados del Censo de 2018 sugieren una reducción en la población de Cali en los últimos 13 años, no es probable que la población en Cali haya disminuido en los últimos años. En efecto, los datos del DANE sobre el número anual de nacimientos vivos y del número de decesos desde 2005 a 2018 indican que la población en 2018 debería estar alrededor de 2.3 millones ^6^. Así mismo, los datos de las afiliaciones al sistema de salud también sugieren que la población en Cali continuo creciendo desde el 2005 ^7^. Debido al crecimiento esperado de la población en la última década en Cali y la evidencia de estas otras fuentes de información, es posible que los resultados del Censo 2018 se deban a problemas en su implementación lo que resultó en un estimado sesgado de la población.

Mientras el DANE analiza las razones por las cuales se obtuvo una población mucho menor de la esperada para Cali, así como en otras ciudades del Valle del Cauca y en otros departamentos del país, los investigadores y tomadores de decisiones seguirán esperando ansiosos los denominadores adecuados para utilizar en sus análisis. Saber cuáles son los mas apropiados es clave para entender las trayectorias de las enfermedades, así como para evaluar el impacto de las políticas públicas para reducir riesgos en la población.
